# Patients at a high risk of PJI: Can we reduce the incidence of infection using dual antibiotic-loaded bone cement?

**DOI:** 10.1186/s42836-022-00142-7

**Published:** 2022-09-07

**Authors:** Christof Berberich, Jerôme Josse, Pablo Sanz Ruiz

**Affiliations:** 1 Department of Medical Training, Heraeus Medical GmbH, 612173 Wehrheim, Hessen Germany; 2 Institut Des Sciences Pharmaceutiques Et Biologiques de Lyon (ISPB), International Center for Research in Infectiology Inserm U1111, CNRS UMR5308, ENS de Lyon, UCBL1, 69008 Lyon, France; 3 Department of Traumatology and Orthopedic Surgery, General University Hospital Gregorio Marañon, Madrid, Spain; 4 Faculty of Medicine, Complutense University of Madrid, Madrid, Spain

**Keywords:** Single low-dose antibiotic-loaded bone cement, Dual high-dose antibiotic-loaded bone cement, Infection prophylaxis, Prosthetic joint infection, Risk factors for infection

## Abstract

Prosthetic joint infection (PJI) is one of the most devastating complications of orthopedic surgery. However, not all patients are equally at the risk of severe infection. The incidences of PJI vary with the host and surgery-related risk factors. It is now generally accepted that some important medical comorbidities may predispose the patients to a high risk of PJI. Time-consuming and invasive surgical procedures, such as revision arthroplasties, are also associated with a high incidence of PJI, presumably due to the increased risk of surgical site contamination. Effective infection-preventing strategies should begin with identifying and optimizing the patients at a high risk of infection prior to surgery. Optimizing the operating room environment and antibiotic prophylaxis are also essential strategies that help minimize the overall incidence of infection in orthopedic surgery. The ideal antibiotic prophylaxis is still under debate, and discussions have emerged about whether variations or adjustments to the standard protocol are justified in patients at a high risk of infection. This also includes evaluating the possible benefits and risks of using high-dose dual antibiotic-loaded bone cement instead of low-dose single antibiotic-loaded bone cement in arthroplasty. This review summarizes the evidence showing that the combination of two local antibiotics in bone cement exerts a strong and longer-lasting antimicrobial effect against PJI-associated pathogens. This conclusion is consistent with the preliminary clinical studies showing a low incidence of PJI in high-risk patients undergoing cemented hemiarthroplasty, cemented revision, and primary arthroplasty if dual ALBC is used. These results may encourage clinicians to consolidate this hypothesis in a wider clinical range.

## Introduction

Prosthetic joint infection (PJI) remains one of the most feared complications of arthroplasty. A growing body of evidence indicates that the risk of PJI development is significantly influenced by patient- and surgery-related factors. Several important host comorbidities have been identified as important conditions that may predispose a patient to PJI. Revision arthroplasty and hemi-arthroplasty in patients with femoral neck fractures are also associated with a higher infection rate than the usual arthroplasty due to longer operative time or more debilitating host health conditions, or both.

Undoubtedly, preoperative patient optimization, strict compliance with hygiene rules in the theatre, and a perioperative antibiotic prophylaxis regimen are the main pillars of infection prevention [1, 2]. However, opposed to the PJI treatment where surgical algorithms take into consideration individual patient and pathogen factors, a similar concept of infection prevention is still lacking. A question of particular interest is whether certain patient groups at a high risk of infection may benefit from more individualized antibiotic prophylaxis instead of a “one-size-fits-all” strategy. Indeed, some evidence supports the hypothesis that the patients at a high risk of infection may benefit from a longer-term perioperative antibiotic prophylaxis. However, its wider clinical practice remains controversial. Many surgeons decide to also use local prophylaxis with antibiotic-loaded bone cement (ALBC) as a complementary tool to systemic antibiotic prophylaxis in arthroplasty. The rationale behind this combined strategy is to create an added antimicrobial defense line in the joint space itself, without exposing the patient to the major risk of toxic side effects. Several registry studies provided evidence showing that the revision rates are reduced if systemic and local antibiotic prophylaxis are combined [3–6]. Given the significantly strong antimicrobial effect of high-dose dual ALBC compared to low-dose single ALBC, it seems that the surgeons have also started to assess its effect in the clinical setting. In this review, we aimed to summarize the available in vitro and in vivo evidence that supports the notion that patients at a high risk of infection may benefit from the additional use of dual ALBC.

### Risk factors for PJI and the idea of a risk-tailored local antibiotic prophylaxis with the aid of dual ALBC

Arthroplasty patients have different PJI risk profiles depending on the type of major comorbidities and the type of surgery. These patient factors increase the risk of infection but their percentage contributions are subject to the individual parameters. Several authors have found that body mass index (BMI) (in particular ≥ 35–40 kg/m^2^), uncontrolled diabetes, malnutrition, wound dehiscence, previous surgical site infections, and previous surgery were the dominant risk factors associated with a two- to five-fold higher incidence of PJI than “normal” [7–9]. Other major predictors of a higher PJI incidence include male gender, diabetes *per se*, post-traumatic arthritis, patellar resurfacing, and discharge to convalescent care [10]. These somehow heterogeneous findings may also explain why recently proposed PJI risk calculator tools [11] are still in the experimental stage. Nevertheless, these discussions serve to sensitize surgeons about the impact of the risk factors of PJI. In the future, they may also help implement a simple risk stratification algorithm in a given hospital instead of frequently encountered “intuition”-based handling of individual risk patients. Some specific procedures like revision surgery and hemi-arthroplasty are repeatedly described to be associated with several folds of high incidence of PJI. It is assumed that this is due to the longer operative time and the more invasive nature of revision procedures as well as to the surgeries on fragile patients in hemi-arthroplasty [12, 13].

Based on these observations, it has been hypothesized that a more risk-tailored antibiotic prophylaxis regimen may help mitigate the higher burden of PJI in these procedures. In principle, there are three options for modification of the standard antibiotic prophylaxis protocol: (1) extending the postoperative duration of antibiotic administration, *e*.*g*. beyond 24 h [14, 15]; (2) adding a second antibiotic to the standard perioperative systemic antibiotic prophylaxis regimen (*e*.*g*. vancomycin or teicoplanin to a cephalosporin) [16, 17]; and (3) in situ implant fixation with high doses and combinations of local antibiotics [18].

**Fig. 1:**
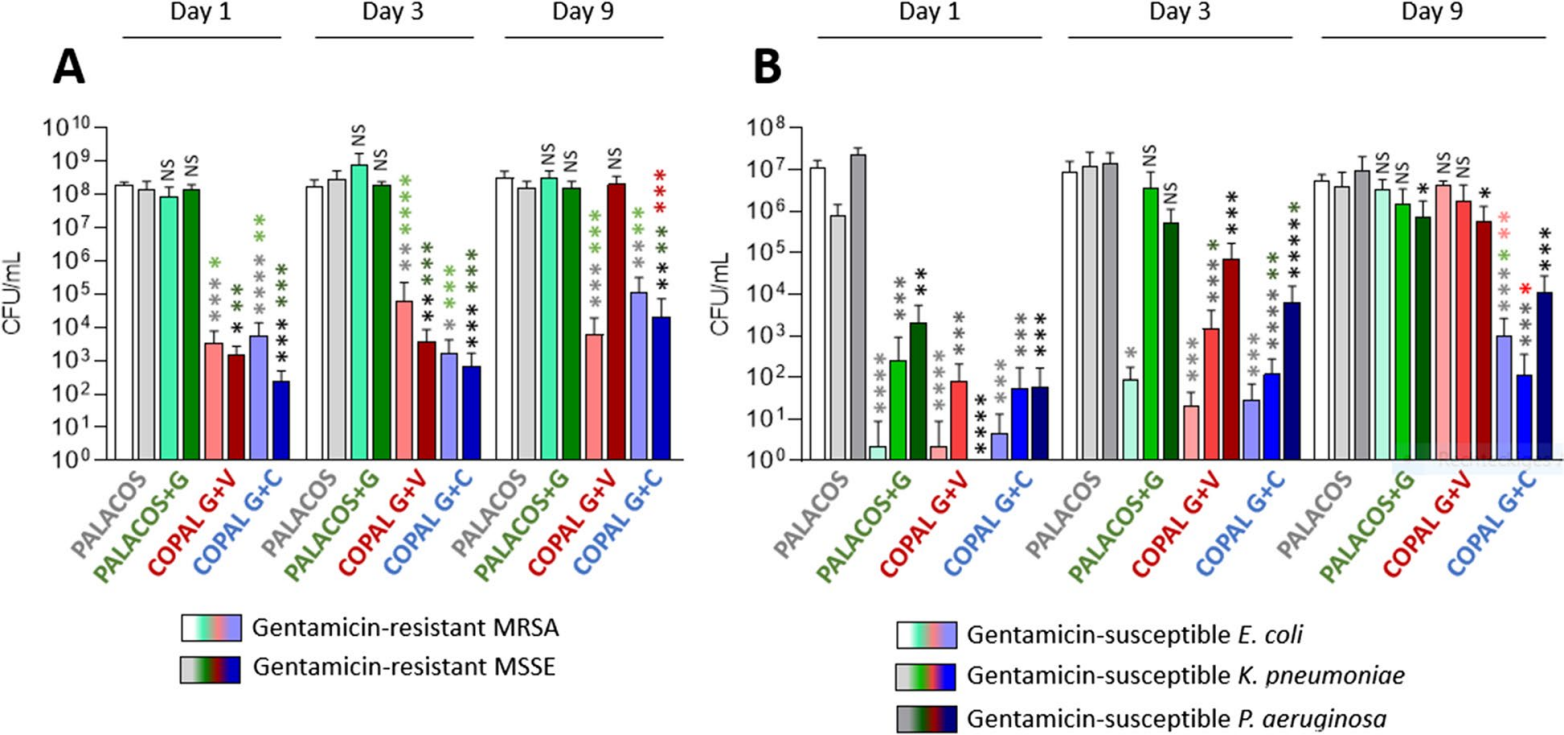
Prophylactic anti-biofilm effect of ALBCs against gentamicin-resistant *staphylococci *(**A**) and gentamicin-susceptible Gram-negative bacteria (**B**). Results were presented as number of colony-forming units (CFU) per mL that are obtained after 24 h of biofilm formation in elution solutions from plain cement and ALBCs that were incubated in bacterial culture medium for 1 day, 3 days and 9 days. In panel **A**, white, light green, light red and light blue histograms correspond to the results for the gentamicin-resistant MRSA strain (Methicillin-resistant *Staphylococcus aureus*); gray, dark green, dark red and dark blue correspond to the results for the gentamicin-resistant MSSE strain (Methicillin-susceptible *Staphylococcus epidermidis*). In panel **B**, white, light green, light red and light blue histograms correspond to the results for *Escherichia coli* strain; mid gray, mid green, mid red, mid blue correspond to the results for *Klebsiella pneumoniae* strain; gray, dark green, dark red and dark blue correspond to the results for the *Pseudomonas aeruginosa* strain. All Gram-negative strains are susceptible to gentamicin. A Dunn’s multiple comparisons tests were performed as follow up test. For each day, *, **, *** or **** mean *P* < 0.05, *P* < 0.01, *P* < 0.001 and *P* < 0.0001 respectively. NS means non-significant in comparison with plain cement. For more details regarding the method, please refer to related reports [24, 25]

Although several reports from single study centers supported options 1 and 2 [14–17], neither tested modifications nor add-ons to the standard perioperative prophylaxis regimen show consistent advantages in a wider range of clinical studies so far [19]. Doubts remain as to whether the benefits outweigh the risks of extended antibiotic administration and antibiotic-mediated systemic side effects when two drugs are used in combination [20]. Therefore, some clinicians focus on reinforcement of the local antibiotic prophylaxis using dual ALBC, assuming a more powerful and more long-lasting antimicrobial effect in situ. Another advantage of this strategy is that the risk of toxic systemic levels is largely avoided if certain concentration thresholds are not exceeded [21, 22]. The idea of a reinforced local prophylaxis is based on the findings from laboratory studies. Dual ALBC loaded with combinations of gentamicin (G) and clindamycin (C) or combinations of gentamicin (G) and vancomycin (V) allow a high mutual release of antibiotics from the carrier matrix [23, 24]. It leads to a stronger and more long-lasting antimicrobial inhibition compared to a single low-dose antibiotic cement. Only the combinations of G + C and G + V in commercial bone cement brand COPAL^®^ (Heraeus Medical, Wehrheim, Germany) were capable of preventing microbial growth of methicillin- and gentamicin-resistant *staphylococci* strains [25]. In contrast, gentamicin alone containing single ALBC brand PALACOS^®^ + G (Heraeus Medical, Wehrheim, Germany) fails to do so. COPAL^®^G + C is superior to COPAL^®^G + V in bacterial inhibition (Fig. 1). These findings are clinically relevant given the increase in antibiotic resistance of these most relevant pathogens in PJI [26]. A recent report published by the same study group further described the antimicrobial effects of G + C and G + V combinations against Gram-negative bacteria, which are also described as important PJI pathogens. COPAL^®^G + C cement was found to exert the strongest antimicrobial effect against gentamicin-susceptible *Escherichia coli*, *Pseudomonas aeruginosa* or *Klebsiella pneumoniae*, presumably due to high concentration and elution of gentamicin in this dual ALBC combination (Fig. 1b) [27].

In addition to the commercial dual ALBC COPAL^®^G + C and COPAL^®^G + V brands, there are widespread non-standardized, off-label, and surgeon-directed antibiotic-cement mixtures in clinical use. Reasons for manually mixing antibiotics into bone cement include economic considerations, the lack of specific pre-mixed ALBC, limited local regulatory approvals, and the need to use specific customized solutions in septic revision arthroplasty. However, systematic biofilm inhibition data or clinical outcomes for such in-theatre admixed cement are not available. So, this review is limited to data compared to the commercial products.

### Clinically meaningful stronger prophylactic effect of dual ALBC (COPAL^®^G + C)

Based on these promising in vitro data, the hypothesis of whether the dual ALBC protects high-risk patients better from infection than single ALBC was recently studied in clinical practice.

#### Results from cemented hemi-arthroplasty patients

**Fig. 2:**
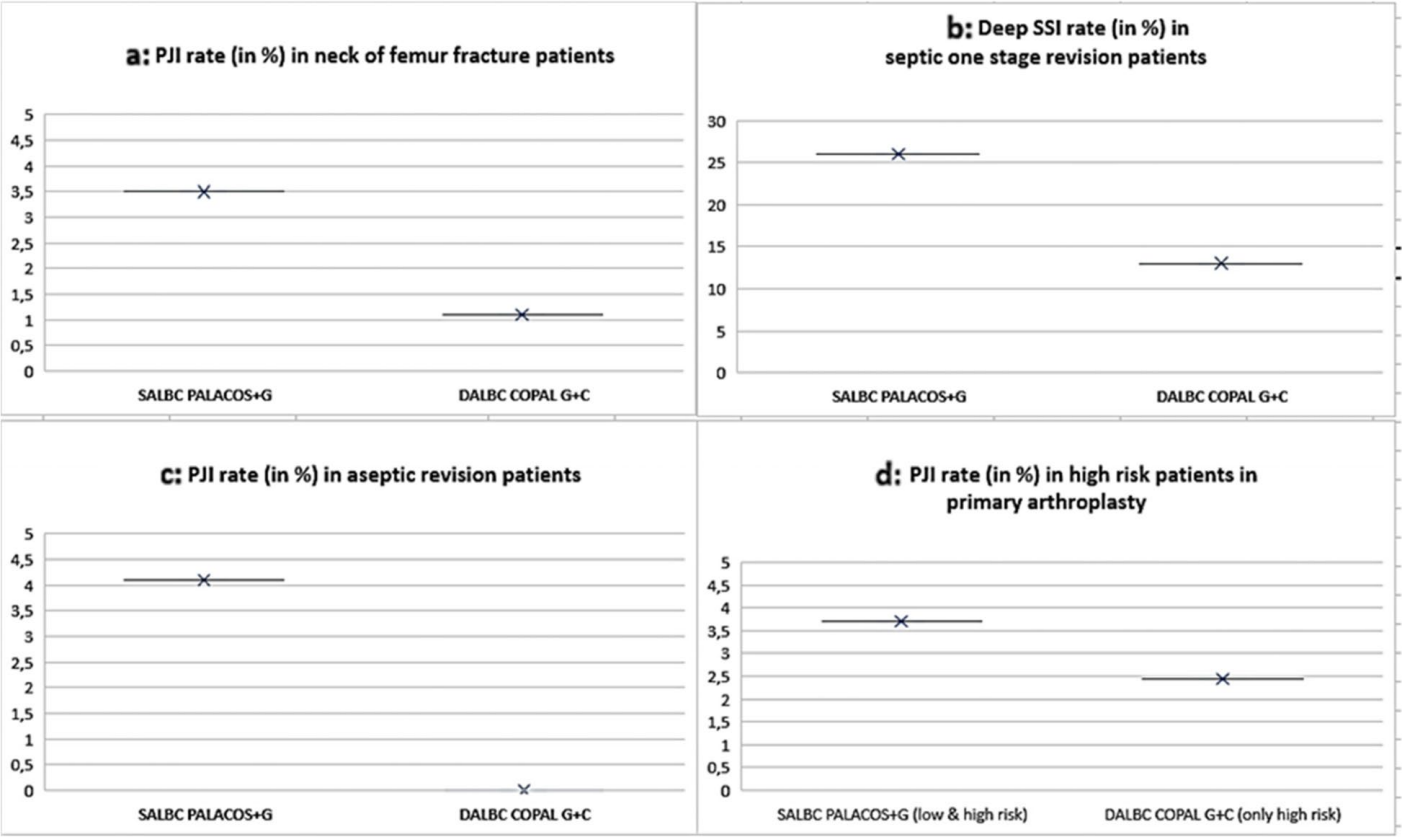
Summary of clinical study results comparing the PJI rate in risk patients after use of single ALBC (SALBC = PALACOS + G) *vs*. dual ALBC (DALBC = COPAL G + C) across different indications. For more details see publications [27, 29–31]

In 2016, a proof of concept first showed that this strategy might positively impact on the incidence of surgical site infection and deep implant infection based on a quasi-randomized clinical trial including 848 femur fracture patients treated with cemented hemi-arthroplasty in the NHS Hospital Trust in the United Kingdom [28]. In this study, the patients were treated with either a single low-dose gentamicin cement (SALBC = PALACOS^®^ + G) representing the current standard of care or dual high-dose antibiotic cement (DALBC = COPAL^®^G + C) as controls. They found the deep infection rates were significantly reduced from 3.5% (SALBC group) to 1.1% (DALBC group) (Fig. 2a). The rates of complications and side effects were similar in both groups. If we also consider the patients with superficial surgical site infection in both groups, the difference between the groups is even more significant (1.7% in DALBC group *vs.* 5.3% in DALBC group) [28]. As a result of these findings, a multi-center, multi-surgeon, parallel, two-arm, randomized clinical trial (WHiTE 8 COPAL study) of approximately 5000 patients is now underway to consolidate the initial findings at a broader clinical level [29].

#### Results of septic *vs*. aseptic cemented revision

Septic revision: Jenny et al. [30] recently performed a retrospective study based on 171 patients undergoing one-stage septic revision. They found that DALBC (COPAL^®^G + C) cement was significantly associated with a lower risk of deep surgical site infection than SALBC (PALACOS^®^ + G) two years after surgery ( 13 *vs.* 26%; odds ratio, 0.42)( Fig. 2b).

Aseptic revision: Sanz-Ruiz et al. [31] retrospectively reviewed 246 patients undergoing aseptic knee revision arthroplasty in a major university hospital in Madrid, Spain. One year after surgery, they found PJI a rate of 4.1% in 103 patients using SALBC (PALACOS^®^ + G) and 0% in 143 patients using DALBC (COPAL^®^G + C), respectively (Fig. 2c). The authors concluded that the use of DALBC was a more potent and a cost-effective method for preventing infection [31].

#### Preliminary results from high-risk patients undergoing cemented primary arthroplasty

**Fig. 3:**
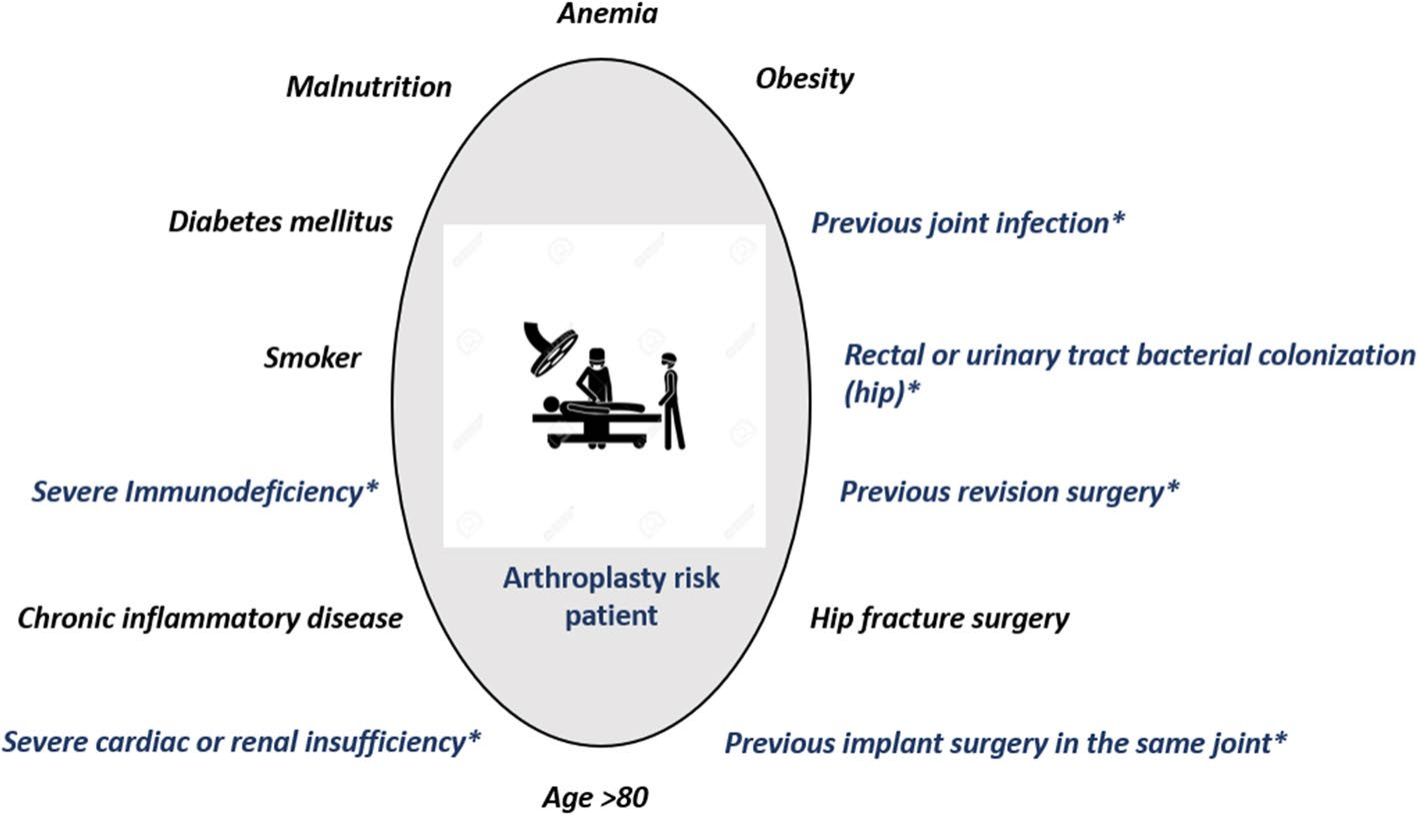
Major patient comorbidities and surgery-related risk factors according to the experiences at the University Hospital Gregorio Maranon, Madrid, Spain [31]. Patients were classified as risk patients if presenting with a factor marked with (*) or with a combination of at least two (knee arthroplasty) or three other factors (hip arthroplasty)

Sanz-Ruiz et al. [32] retrospectively studied 2,551 patients undergoing cemented primary arthroplasty between 2015 and 2018. Preoperatively, the patients were assessed based on the institution-specific patient risk algorithm (Fig. 3). In this series, 2,368 patients (92.8%) with low and high risks of infection were treated with SALBC (PALACOS^®^ + G), and 183 patients (7.2%) with an exclusively high risk were treated with DALBC (COPAL^®^G + C). One year after surgery, the PJI rates were 3.7 and 2.45%, respectively (Fig. 2d). Contrary to expectations of a higher PJI rate in the exclusively high risk group they observed a trend to a lower PJI incidence if treated with DALBC.

### Does use of high dose dual ALBC drive more antibiotic resistance?

This critical topic must be considered as surgeons have repeatedly expressed their concern that wider use of ALBC may promote antibiotic resistance. Usually, antibiotics are clinically selected based on the antibiogram that is determined in vitro by the susceptibility or resistance tests of bacteria at different dilutions. For systemic antibiotic therapies, the minimal inhibitory concentration breakpoints are determined by both pharmacokinetic expectation of drug concentration in the tissues and the knowledge on minimal inhibitory concentrations (MIC) of the pathogen in vitro. However, such guidance is not available in local antibiotic therapies. Because the peak concentrations are often 10- to 100-fold higher than those reported in the systemic administration, susceptibility reporting criteria and minimal inhibitory concentration breakpoints are often not applicable to the local therapies [33]. Therefore, bacteria classified as intermediately resistant in the classical antibiogram may remain susceptible to high local concentrations of antibiotics, which is particularly true for antibiotics with bactericidal and strictly concentration-dependent effects, *e*.*g*., aminoglycosides. In view of these facts, induction of antibiotic resistance surrounding ALBC should not be a clinical concern. However, in the presence of a prior high-level aminoglycoside-resistant bacteria, survival and subsequent selection of these organisms may still occur, possibly even leading to recolonization in the carrier matrix [34].

Four recent studies addressed the question of whether the use of ALBC in a larger clinical setting increases bacterial antibiotic resistance in PJI. It was observed that its frequency was comparable in PJI patients regardless of whether their prostheses were cemented with ALBC or not [35–38]. One study went even further and looked at the resistance pattern of the few remaining gentamicin- and clindamycin-resistant bacteria in the PJI patients receiving cemented hemiprostheses with DALBC COPAL^®^G + C. The resistance to the antibiotics was limited, and there was no cross-resistance to the important therapeutic antibiotics, such as rifampin and quinolones [36]. These findings showed that the use of ALBC neither promotes widespread bacterial resistance nor affects the therapeutic options for those antibiotics recommended for the treatment of Gram-positive or Gram-negative pathogens in PJI.

## Conclusions

Patients with multiple comorbidities and patients undergoing hemi- and revision arthroplasties are at a higher risk of developing PJI. Implementation of a risk-adapted antibiotic prophylaxis regimen in those patients is currently under clinical investigation. The use of high-dose DALBC may be an attractive option to decrease the risk of local infection by ensuring high peak concentrations and synergistic antimicrobial effects in situ. The preliminary clinical results support this hypothesis, showing that the incidence of PJI is reduced if the prostheses are cemented with DALBC in patients at high risk of infection. These benefits are not associated with more systemic side effects or high antimicrobial resistance. Further high-quality clinical studies are needed to consolidate these data on a broad clinical range.

## Data Availability

No own datasets were generated or analyzed during the current study. The referenced data are part of clinical study publications listed in the bibliography.

## Abbreviations

PJI: Prosthetic joint infection
PAP: Perioperative antibiotic prophylaxis
ALBC: Antibiotic-loaded bone cement
BMI: Body mass index
G: Gentamicin
C: Clindamycin
V: Vancomycin
SALBC: Single low-dose antibiotic-loaded cement
DALBC: Dual high-dose antibiotic-loaded cement
